# Does Landscape Fragmentation Influence Sex Ratio of Dioecious Plants? A Case Study of *Pistacia chinensis* in the Thousand-Island Lake Region of China

**DOI:** 10.1371/journal.pone.0022903

**Published:** 2011-08-04

**Authors:** Lin Yu, Jianbo Lu

**Affiliations:** 1 College of Life Sciences, Zijingang Campus, Zhejiang University, Hangzhou, People's Republic of China; 2 College of Life and Environmental Sciences, Hangzhou Normal University, Hangzhou, People's Republic of China; Institut Mediterrani d'Estudis Avançats (CSIC/UIB), Spain

## Abstract

The Thousand-Island Lake region in Zhejiang Province, China is a highly fragmented landscape with a clear point-in-time of fragmentation as a result of flooding to form the reservoir. Islands in the artificial lake were surveyed to examine how population sex ratio of a dioecious plant specie *Pistacia chinensis* B. was affected by landscape fragmentation. A natural population on the mainland near the lake was also surveyed for comparison. Population size, sex ratio and diameter at breast height (DBH) of individuals were measured over 2 years. More than 1,500 individuals, distributed in 31 populations, were studied. Soil nitrogen in the different populations was measured to identify the relationship between sex ratio and micro-environmental conditions. In accordance with the results of many other reports on biased sex ratio in relation to environmental gradient, we found that poor soil nitrogen areas fostered male-biased populations. In addition, the degree of sex ratio bias increased with decreasing population size and population connectivity. The biased sex ratios were only found in younger individuals (less than 50 years old) in small populations, while a stable 1∶1 sex ratio was found in the large population on the mainland. We concluded that the effects of landscape fragmentation on the dioecious population sex ratio were mainly achieved in relation to changing soil nitrogen conditions in patches and pollen limitation within and among populations. Large populations could maintain a more suitable environment in terms of nutrient conditions and pollen flow, subsequently maintaining a stable sex ratio in dioecious plant populations. Both micro-environmental factors and spatial structure should be considered in fragmented landscape for the conservation of dioecious plant species.

## Introduction

Under natural selection, a 1∶1 ratio has been proven as a theoretical and evolutionarily stable sex ratio; otherwise there would be a frequency-dependent advantage to the rarer sex [1]. This theory has been supported with many theoretical and empirical studies [2], [3], [4], [5]. However, there has been a proliferation of studies describing the existence of biased sex ratios in nature, providing new dimensions to Fisher's central theory [6].

In dioecious plants, most sex ratio studies have attributed sex ratio bias to sex-differentiated mortality in stressful habitats. Females in dioecious plant populations often invest more in reproduction and less in growth and maintenance than males. Females need to allocate energy and nutrients to flowers and fruits, whereas males invest only in flowers [7], [8]. This differential investment between sexes can result in contrasting survival rates [9] and distinct growth patterns [10], [11]. Female plants can suffer higher mortality in stressful habitats, such as in nutrient-deficient soil, locations with strong competition from other plants, or in climatically stressed environments [12]. Sex-biased herbivory might also create a biased sex ratio [13], [14], [15]. Other studies of progeny sex ratios have observed that pollen competition might produce different sex ratios in offspring [16]. High stigmatic pollen loads can induce pollen tube competition and produce stronger female-biased seed sex ratios [17].

Land clearing and environmental destruction by humans have led to the subdivision of originally continuous habitats into smaller, more isolated patches, which influence more plant populations and communities all over the world [18], [19]. Effects of habitat fragmentation are complex and include: localized micro-climates and environmental change [20], [21], [22], [23], increased risk of local extinction, disrupted dispersal, habitat deterioration due to edge effects, and increased risk of invasions from exotics [24], [25]. In fragmented landscapes, pollination rates can be influenced by population size and population density [26], [27], [28], and the reduced amount of compatible pollen might also induce pollen limitation in small populations [29]. Dioecious plants are expected to be more sensitive to change in population size and structure than self-compatible species and thus also more sensitive to habitat fragmentation [30], [31].

However, biased sex ratio variation in dioecious plants has seldom been studied in naturally fragmented landscapes. The fact that fragmentation would induce micro-environment change and deterioration as well as pollen limitation for small populations has already been established, and it has also been shown that micro-environment gradients and pollen limitation can lead to a biased sex ratio in dioecious plants [32], [33], [34], [35]. However, evidence is lacking on whether a biased sex ratio might be mediated by micro-environment change or pollen limitation due to landscape fragmentation in dioecious plants.

The objective of this study was to examine how the micro-environment gradient and population structure influence sex ratio in a fragmented landscape. It was expected that biased sex ratios were most severe where pollen flow was limited in the populations, or the micro-environment nutrient conditions were poor. The Thousand-Island Lake, an artificial reservoir formed 50 years ago, contains more than 1,000 islands of different sizes. A clear fragmentation point-in-time, a high degree of fragmentation and protection from anthropogenic disturbance make it as perfect natural laboratory, providing a good study site both in terms of spatial and time scales.

*Pistacia chinensis* B. is a wind-pollinated, perennial dioecious plant specie, which has been found on many islands in the Thousand-Island Lake [36]. As a perennial specie, some *P. chinensis* individuals have existed both before and after fragmentation, making it possible to search for evidence of sex ratio variation 50 years after fragmentation.

In this study, more than 30 populations of *P. chinensis* of different sizes were investigated. Sex ratios, spatial structure and soil nitrogen of populations on the islands were compared with a large population on the mainland near the lake. The specific questions asked were: (1) how much variation is in the sex ratio in natural *P. chinensis* populations? (2) Does soil nitrogen influence the sex ratio of the populations on the islands? (3) Compared to the large population on the mainland, do small and isolated populations display a higher variability in the sex ratio? (4) Are there sex ratio differences among different age classes of individuals in the populations?

## Materials and Methods

### Study Specie

*Pistacia chinensis* B. is a deciduous, wind-pollinated, large shrub or small tree, which grows to 5 m, with alternate, predominantly paripinnate leaves. Male and female flowers mature in March and April, and seeds are produced in September [37]. Sex determination mechanisms in this specie are still unknown. The sex of the plant is unrecognizable until it flowers. Nursery-grown *P. chinensis* plants first flower at 6–10 years of age; however, wild trees appear to grow and reach flowering stage more slowly [38]. Males always mature earlier than females [39]. Vegetative reproduction does not occur naturally in this species [37].

*P. chinensis* is native to a broad region from Afghanistan to China, and to the Philippines. Because of the high quality of wood and high oil content of seeds (50%) [40], *P. chinensis* has been widely planted and exploited in China for timber [41] and diesel oil [40].

### Study Site

The Thousand-Island Lake is an artificial lake located in Chun'an County, western Zhejiang Province, China. The resevoir was created in 1959 as a result of the construction of the Xin'an River Hydroelectric Power Station. The Thousand-Island Lake name reflects the 1,078 islands in the lake with areas larger than 2500 m^2^. The lake covers 60 km from east to west, and 50 km from north to south. The surface area of the lake is 583 km^2^, with a total island area of 409 km^2^. Forest covers 68% of the island surface area. The Thousand-Island Lake was identified as a national scenic area by the State Council in 1982 and it is now major national scenic location and the largest national forest park.

**Figure 1:**
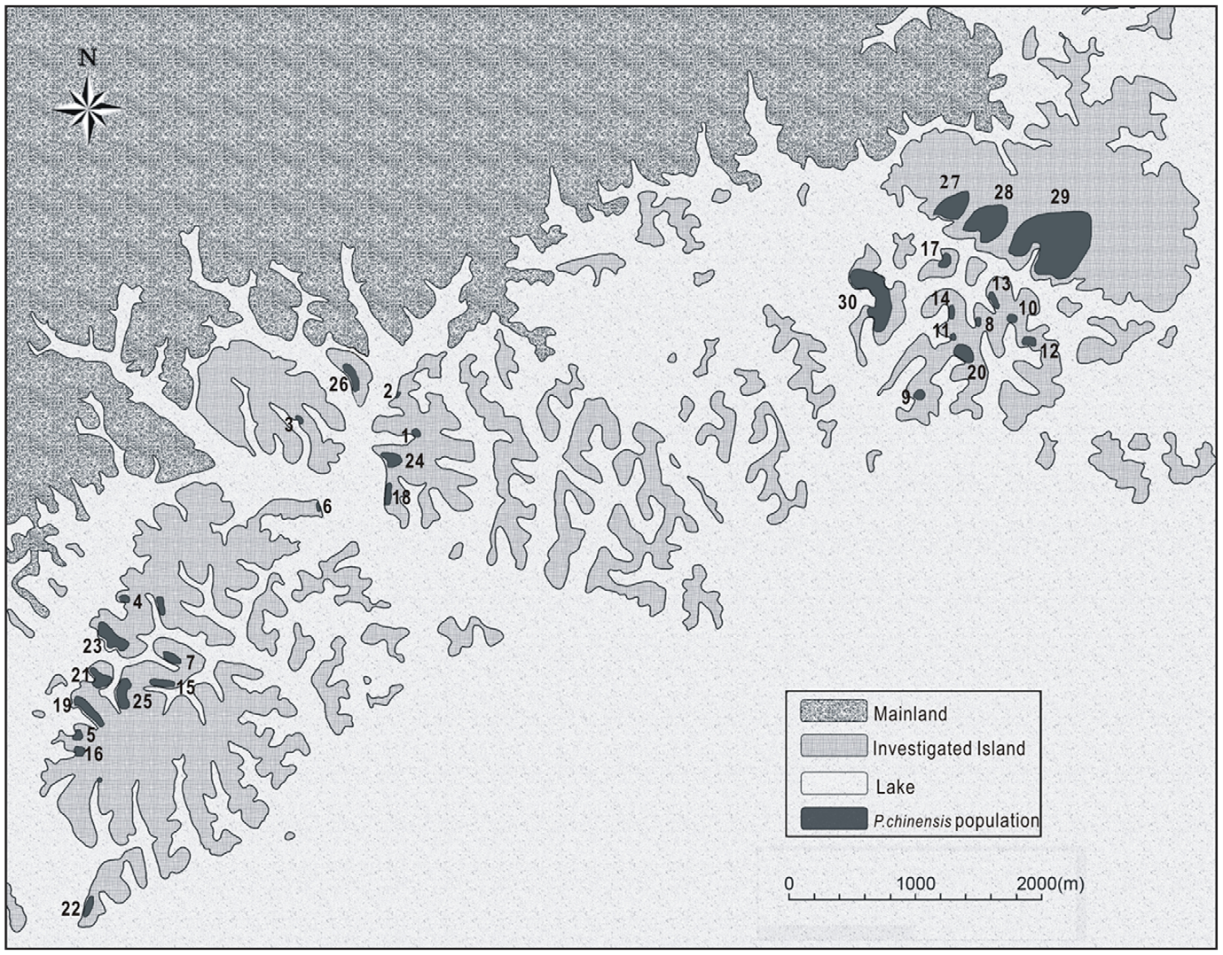
Map indicating the islands surveyed in the study and locations of 30 *P. chinensis* populations on the islands.

We investigated more than 60 islands (29°23′–29°35′N, 118°52′–118°56′E) (Fig. 1) in 2008 and 2009, at the north end of the Thousand-Island Lake, covering an area of 80 km^2^. A persistent population of *P. chinensis* on the mainland (10 km from the lake) was also investigated as a control. The landscape pattern of the survey area underwent a drastic change after the formation of the reservoir. The water level was raised 108 meters, the tops of mountains formed islands and peninsulas. Benefiting from a policy of forest protection, the Thousand-Island Lake region has been well protected since the establishment of the reservoir in 1959, and provides an adequate case for the study of fragmentation.

### Data Collection

Field data were collected during the flowering and fruiting season from March 2008 to November 2009 on 60 islands in the Thousand-Island Lake. Only on 10 islands there were found 1016 individuals (diameter >5 cm) in 30 populations. In the continuous population on the mainland, 290 individuals in 5 plots (50 m ×50 m) were investigated. It was estimated that there were about 500 individuals in the mainland population. The presence of male or female flowers and seeds was used to determine the sex of every individual on the surveyed islands and in each of the plots within the mainland population. Each individual was recorded as one of three categories: male, female or indeterminate, and marked with a gender and number. Diameter at breast height (DBH) was measured for each individual.

We digitized a 1∶10000 map of the Thousand-Island Lake region using ARCGIS (9.3) software, and marked the exact locations of *P. chinensis* populations on the digital map. Distances between *P. chinensis* populations in the islands were measured in ARCGIS.

### Age Calculation

The age of each individual was estimated by DBH to explore different responses to fragmentation by age. According to research on growth in *P. chinensis* [39], the fast growth stage appears during the first 15 years, during which the average annual growth is 0.35 cm. After 40 years, growth declines slowly. Using the current annual growth curve of *P. chinensis* [39], we could calculate the age of specimens by DBH.

### Soil Nitrogen

To estimate the micro-environment conditions for *P. chinensis* populations, soil nitrogen was used as an indicator. Soil samples were collected from *P. chinensis* populations with population sizes larger than 10 (in populations smaller than 10, individuals always separate from one another, making it difficult to use soil samples to reflect micro-environmental quality). In March 2009, five points were randomly selected within each population to collect a soil sample. Soil samples were collected with a metallic corer (depth: 50 cm, diameter: 5 cm) at each site. The soil obtained in all cases was air-dried and sieved (0.5 mm mesh) and total nitrogen concentration (mg N per g soil) was determined by the Kjeldahl technique (Kjeltec 2200) [42].

### Statistical Analysis

Chi-square and Fisher exact probability test for 2×2 contingency were used to test the deviation of the sexes in each location from mainland population. The influences of age and population size to the probability of sex were tested by binary logistic regression, age was set 0 or 1 (0, younger than 50 years; 1, older than 50 years) in statistic analysis to perform the influence of fragmentation. The Omnibus test was used to test the model coefficients. Pearson product-moment correlations were used to check if population size, soil nitrogen and patch connectivity index had effects on population sex ratio (female/male). Population size was log-transformed prior to analyses in order to meet normality and homoscedasticity, though log-transformation performs poorly in statistic analysis when dealing the count data with zero observation [43], there were no zero data for population size in our study. The analyses were conducted using SPSS (15.0) software.

We tested whether the sex ratio correlated with connectivity [44] of each *P. chinensis* population. The measure of patch connectivity used here is a negative exponential dispersal kernel.
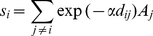
(1)


The indices *i* and *j* refer to the focal and surrounding *P. chinensis* populations. Where *S_i_* is the connectivity of the patch, *d_ij_* (m) is the nearest edge-to-edge distance between patches *i* to *j*. *A_j_* is the area of the patch (transformed into population size). Parameter *a* scales the effect of distance to migration (1/ *a* is the average migration distance). (a) was a measure originally used in the Incidence Function Model (IFM) [45]. In our study, pollen limitation was the most significant factor influenced by connectivity. Since *a* is not known for *P. chinensis*,concerning the researches of pollen dispersal distance for wind-pollinated species, such as *Quercus robur* (22.1–58.41 m) [46], *Quercus petraea* (18.41–64.56 m) [46], *Fagus silvatica* (50 m) [47], *Pinus densiflora* (68 m) [48], *Pinus sylvestris* (83 m) [49], *Araucaria angustifolia* (83 m) [50], *Pinus flexilis* (140 m) [51], and *Fraxinus excelsior* (328 m) [52]. We suppose that setting *a*  = 0.01(1/*a* = 100) is more suitable than 1, 0.1 and 0.001 for the estimation of pollen dispersal distance of *P. chinensis* in our research.

## Results

The flowering sex ratio was determined for 1,306 individuals, including 686 males, 527 females and 93 individuals for that sex was indeterminate. These included 1,016 individuals from 30 populations on the islands, and 290 individuals from 5 plots in the continuous population on the mainland. There was no flowering female found in 3 small populations. The sex ratio of mainland population was approximately 1∶1, while male individuals were superior in more than 85% island populations, only 4 of 30 island populations have more female individuals (Table 1). 2×2 contingency test was used to compare female and male numbers of each island population with which of mainland population. A statistically significant male bias (chi-square, *χ^2^*>3.84; fisher exact test, *P*<0.05) of small island populations individuals compared to the mainland population, similar result was not found in big island populations (Population size >100) (Table 1).

**Table 1:** Population size, number of males, number of females, sex ratio (Female/Male), Pearson value of *Chi*-squared and *P* value of Fisher exact test in 2×2 contingency test compared sex ratio of each location with mainland are given.

Populaotion	GPS	Size	Males	Females	F/M	Pearson Value	FET *P*
1	29°34′43″N 118°53′41″E	5	5	0	0.00	-	0.06
2	29°34′37″N 118°53′45″E	5	4	1	0.25	-	0.37
3	29°34′40″N 118°53′29″E	5	4	0	0.00	-	0.12
4	29°34′02″N 118°52′54″E	5	2	1	0.5	-	1.00
5	29°33′50″N 118°52′46″E	5	4	1	0.25	-	0.37
6	29°34′27″N 118°53′24″E	7	6	1	0.17	-	0.12
7	29°34′01″N 118°53′04″E	7	7	0	0.00	-	0.01*
8	29°34′54″N 118°55′25″E	7	4	3	0.75	-	1.00
9	29°34′43″N 118°55′15″E	8	7	1	0.14	-	0.07
10	29°34′55″N 118°55′32″E	8	5	3	0.60	-	0.72
11	29°34′52″N 118°55′21″E	10	6	3	0.50	-	0.50
12	29°34′51″N 118°55′35″E	10	7	2	0.29	-	0.17
13	29°34′58″N 118°55′27″E	11	6	4	0.67	-	0.75
14	29°34′58″N 118°55′21″E	15	10	5	0.50	2.29	0.29
15	29°33′58″N 118°53′04″E	18	10	4	0.40	1.45	0.17
16	29°33′28″N 118°52′52″E	18	14	2	0.14	8.21**	0.00**
17	29°35′04″N 118°55′20″E	21	8	9	1.13	0.09	0.80
18	29°34′27″N 118°53′41″E	24	14	9	0.64	0.87	0.39
19	29°33′51″N 118°52′49″E	30	20	7	0.35	5.38*	0.03*
20	29°34′49″N 118°55′23″E	30	16	11	0.69	0.72	0.42
21	29°33′58″N 118°52′49″E	33	16	12	0.75	0.42	0.55
22	29°33′25″N 118°52′48″E	35	19	10	0.53	2.30	0.17
23	29°34′09″N 118°52′48″E	35	15	17	1.13	0.17	0.71
24	29°34′33″N 118°53′41″E	42	19	16	0.84	0.16	0.72
25	29°33′55″N 118°52′54″E	44	20	17	0.85	0.14	0.73
26	29°34′43″N 118°53′35″E	56	33	15	0.45	5.34*	0.03*
27	29°35′13″N 118°55′20″E	61	24	34	1.42	1.67	0.25
Population Size <100		555	305	188	0.62	9.01**	0.00**
28	29°35′09″N 118°55′26″E	136	74	54	0.73	1.76	0.20
29	29°34′04″N 118°55′31″E	162	75	84	1.12	0.51	0.49
30	29°34′59″N 118°55′07″E	163	92	65	0.71	2.49	0.13
Population Size >100		461	241	203	0.84	0.86	0.36
Total Island Populations		1016	546	391	0.72	4.94*	-
Mainland	29°42′39″N 119°03′27″E	500	140	136	0.97	-	-

“*”Chi-square *χ2*>3.84, *df* = 1,P<0.05. and Fisher exact test P<0.05.

“**”Chi-square *χ2*>6.635, *df* = 1,P<0.01. and Fisher exact test P<0.01.

**Figure 2:**
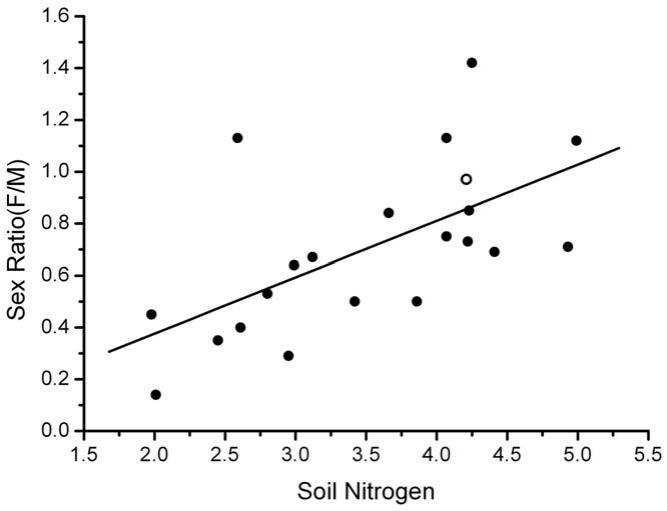
Correlation of soil nitrogen and sex ratio (F/M) of 20 populations on the islands (closed circle) and one population on the mainland (open circle), The Pearson correlation coefficient *r* = 0.615, *P* = 0.003.

Soil nitrogen content could reflect the nutrient conditions of the micro-environment. We obtained 100 soil samples from 20 *P. chinensis* populations on the islands with more than 10 individuals, and 10 from a large continuous population on the mainland. Soil nitrogen for the large population on the mainland (4.21 mg/g) was higher than most of the populations on the island (average 3.84 mg/g). Sex ratios (F/M) of each location were proved to be positively correlated with soil nitrogen by pearson correlations (Fig. 2). The trend was expected under the hypothesis of female and male competition in stress environments. A male-biased sex ratio correlated with lower content of nitrogen in the soil where *P. chinensis* grew.

**Figure 3:**
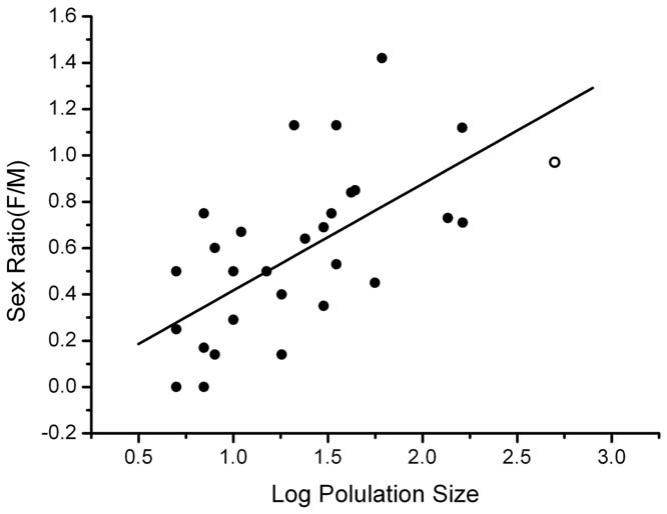
Correlation of log population size and sex ratio (F/M) of 30 populations on the fragmented islands (closed circle) and one population on the mainland (open circle), The Pearson correlation coefficient *r* = 0.661, *p*<0.001.

**Figure 4:**
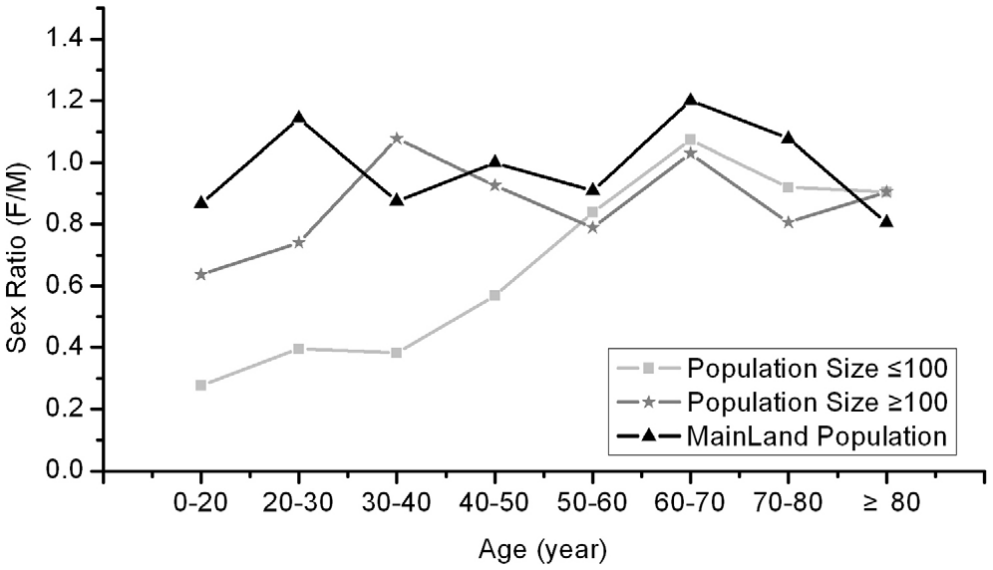
Sex ratio (F/M) of the mainland population (triangle), 3 populations with more than 100 individuals (star), and 27 populations less 100 individuals (diamond), by age class.

The correlation of the sex ratios (F/M) of 31 populations and population size was found under the hypothesis of pollen limitation (Fig. 3). Using the DBH of every individual, we calculated the age for each, and classified the individuals into 8 age classes. We found a significant decline of sex ratio in 4 age classes less than 50 years old. This phenomenon only existed in small populations (less than 100 individuals), while individuals older than 50 years displayed the theoretical 1∶1 sex ratio (Fig. 4).

Binary logistic regression models were used to detect the influences of population size and individual age to the proportion of sex. Age was set as 0 or 1 (“0”, younger than 50 years; “1”, older than 50 years) in the statistic analysis to reflect the effects of 50-years fragmentation. Results of binary logistic regression showed that population size and individual's age strongly affected the proportion of sex in the group of individuals from small island populations (population size <100), whereas sex proportion of big populations (population size >100) and mainland population failed to explain any of variability for the studied variables (Table 2). The results indicated that sex proportion changed significantly after the fragmentation in small island populations, while the sex proportion of large island and mainland populations could maintain a relatively stable sex ratio around 1∶1 through the whole years (Table 2, Fig. 4).

**Table 2:** Probability the sex of *P.chinensis* individuals in dependence of population size and age fitted by binary logistic regression for island populations, and probability of sex for mainland population individuals in dependence of age only.

	Effect	Wald *χ2*	*df*	*P* value	Omnibus Tests		
					*Chi*-squar	*df*	*P* value
Population Size <100	Population Size	12.089	1	0.001	31.356	2	<0.001
	Age	16.077	1	<0.001			
	Constant	43.045	1	<0.001			
							
Population Size >100	Population size	0.75	1	0.386	0.88	2	0.644
	Age	0.105	1	0.746			
	Constant	0.926	1	0.336			
							
Total Island Population	Population size	6.654	1	0.01	18.118	2	<0.001
	Age	7.93	1	0.005			
	Constant	36.693	1	<0.001			
							
Mainland Population	Age	0.091	1	0.763	0.091	1	0.763
	Constant	0.167	1	0.683			

The results of Omnibus Test on regression are given.

In this study, a negative exponential dispersal kernel (a) was used to measure the connectivity of 30 populations on the islands. Since we focused on pollen limitation, the parameter α was set 0.01. This index could reflect the isolation condition of *P. chinensis* populations. Individuals in 20 populations larger than 10 individuals were divided into two groups, those younger and those older than 50 years. We found that the sex ratios of the younger group individuals correlated with the connectivity indicator S, while sex ratios in the older group did not. The populations having higher patch connectivity tended to have higher sex ratio (F/M) in younger group.

## Discussion

Environmental effects on sex ratios have been widely found in dioecious plants [34], [53], [54]. As females in dioecious species usually have lower yearly growth increments of height and stem diameter, and consequently higher mortality due to the higher energetic cost [35], [55], [56], the proportion of female and male specimens could follow the changes in environmental conditions in a particular stage of succession [57], [58]. Our results supported the hypothesis that stressful environments have more negative impacts on females than males. Male individuals were superior in 86% island populations, and the proportion of male individuals increased with decreasing soil nitrogen content (Fig. 2). Pre-fragmentation and post-fragmentation born individuals showed great differences on sex proportions in island populations (Fig. 4, Table 2). We suggest that the deterioration of the micro-environment after fragmentation might lead to such male-biased sex ratios.

Since the dam was built on the Xin'an River in 1959, the micro-environment of the fragmented landscape has changed greatly. The soil nitrogen content of mainland population was detected larger than that found for most of the islands. Edge effects and a decline in biodiversity have also been detected in the Thousand-Island Lake region [59]. Large islands have better preserved plant cover than small islands [59], creating more resistance to water and soil erosion, factors that might result in different soil conditions for *P. chinensis* populations. Males might be more competitive in this changed environment.

The study also documented sex ratio variation with spatial structure of *P. chinensis* populations, where small and isolated patches tended to have more male-biased sex ratios (Table 2, Fig. 3, Fig. 4). In this study, only young individuals (less than 50 years old) presented male-biased sex ratio in small populations on the islands (Fig. 4). Population size could influence the sex proportion only for small populations but not big populations (Table 2). The sex ratio was maintained at approximately 1∶1 in the mainland population, in the 3 large island populations, and also in the older group in small populations (Fig. 3). Therefore, our results proved that big populations of *P. chinenesis* can resistant to the distortion of sex ratio better than small populations.

**Figure 5:**
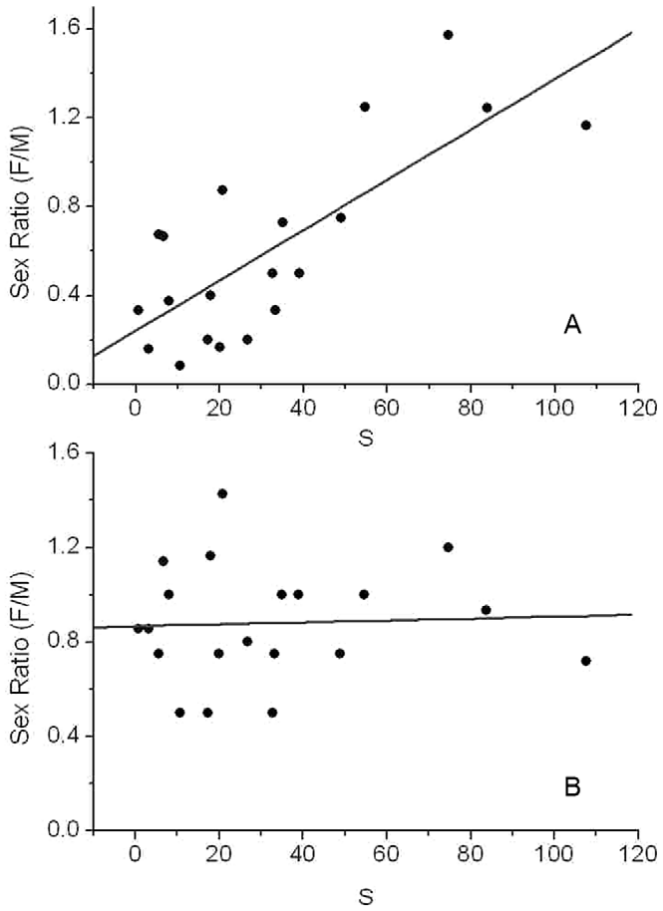
Correlation of patch connectivity index S and sex ratio of individuals (A) younger and (B) older than 50 years in 20 populations of *P. chinensis* on the studied islands, The Pearson correlation coefficient A: r = 0.77, *P*<0.0001; B: r = 0.044, *P* = 0.843.

The same sex ratio pattern was found in the relationship with population connectivity. Sex ratio correlated with population connectivity in individuals younger than 50 years but not in those older than 50 years (Fig. 5). Unfortunately we did not have pre-fragmentation data on populations and patch size of *P. chinensis* in the region; we could not therefore know whether or not the populations with smaller patch sizes were larger before they were submerged. However, our data did show that populations on small or narrow islands were much smaller than on big islands or on the mainland (Fig. 1), and there was a great difference of sex ratio between pre-fragmentation and post-fragmentation born individuals. We suggest there was a mechanism underlying pollen limitation after landscape fragmentation.

In Taylor's study of the dioecious herb Silene alba, sex ratio was shown to be directly influenced by the quantity of pollen. High quantity pollen (pollen mixture from several males) could produce more female progeny than low quantity pollen (pollen from a single male) [16]. Similar results were also found in the dioecious herb Rumex nivalis, where stronger female-biased sex ratio progeny were presented with maternal parents closer to the males due to the pollen tube competition [17]. The amount of pollen captured by stigmas could potentially affect both quality and sex ratio of offspring [17]. In our study, fragmentation could reduce the pollen flow within and among populations, which might lead to fewer female individuals among offspring than before, which might explain the effects of population size and patch connectivity to the population sex ratio after the fragmentation (Table 2, Fig. 5). However, since P. chinensis is a perennial specie, the sex ratio would not change greatly in one generation. Through the influence of several generations, the sex ratio would be regularly distorted to a male-biased ratio from the 1∶1 original sex ratio, and that could be a reasonable hypothesis to explain the slope of sex ratio variation of small populations individuals 50 years post-fragmentation (Fig. 4).

**Figure 6:**
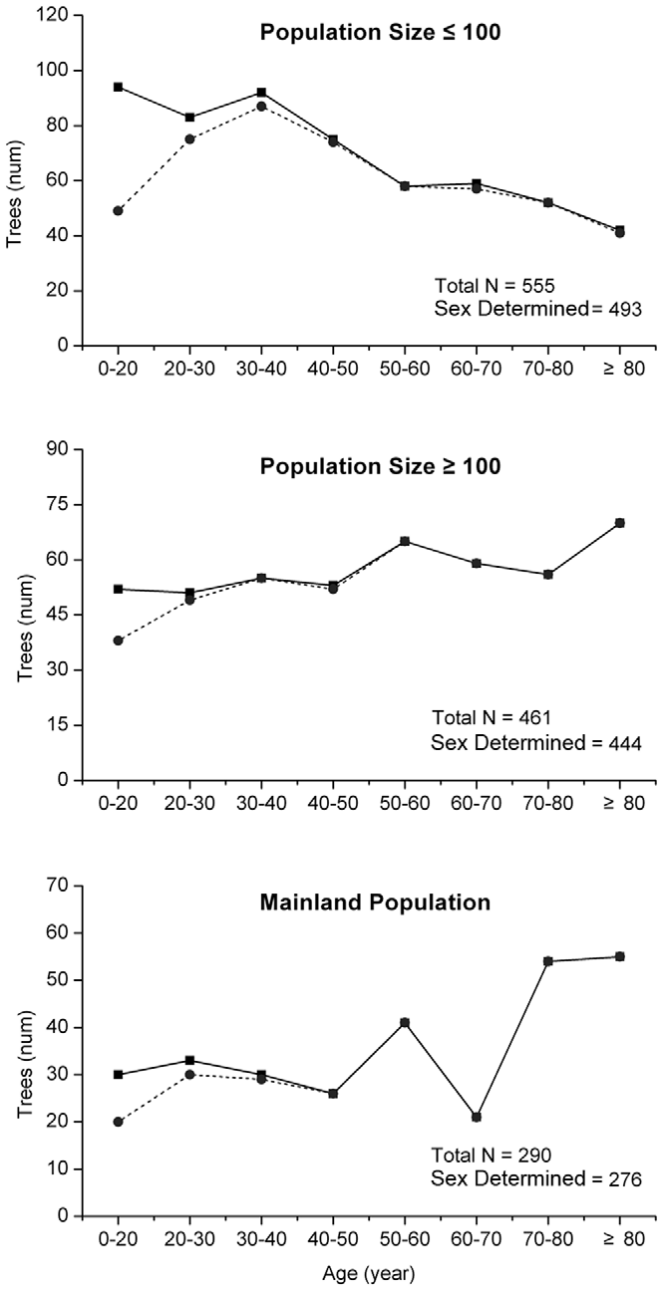
Age structure of mainland population, 3 populations with more than 100 individuals and 27 populations less 100 individuals, investigated individuals (solid line), sex determined individuals (dotted line).

Comparison of continual population with fragmented population is a good way to prove the effects of fragmentation. However, only one natural and continual *P. chinensis* population was found around Thousand Island Lake region, this limit might lead some misjudgement or bias in our conclutions. Nevertheless, big island populations present similar results with the mainland population, which are significant different with small island populations (Table 1, Table 2, Fig. 4), it will partially confirm the influence of fragmentation. Some researches had reported that male begin flowing at smaller size than do females as a result of higher energy costs of reproduction in females than in males [60].This mechanism might explain the male-biased sex ratios in our result. The data showed that unflowering individuals concentrated in younger age classes, in small island populations (less than 100 individuals) nearly 50% individuals were unflowering in the first age class, this proportion dropped to 10% in 20 to 30 years age class (Fig. 6). However, even all the undetermined individuals are females, the sex ratios of small island populations are still significant male-biased (chi-square, *χ^2^*>3.84; fisher exact test, *P*<0.05) in 20 to 30 years (F/M = 30/53) and 30 to 40 years (F/M = 29/63) age classes. We suppose that higher mortality of female individuals and less female in primary sex ratio due to the pollen limitation still might play an important role in the male-biased sex ratio in our research.

Male-biased sex ratio was quite pronounced in small populations on the islands, due to soil nitrogen condition and pollen limitation in small and isolated populations. It might also lead to genetic drift and local extinction for the population [61]. However future studies are needed to detect pollen flow and seed flow among populations at the genetic level, and that would be stronger evidence for the influence of fragmentation on *P. chinensis* populations.
